# Supplementary figures and images for: The indelible toll of enteric pathogens: Prevalence, clinical characterization, and seasonal trends in patients with acute community-acquired diarrhea in disenfranchised communities
Source: PLoS One. 2023 Mar 13;18(3):e0282844. doi: 10.1371/journal.pone.0282844 (PMC10010529; doi:10.1371/journal.pone.0282844)

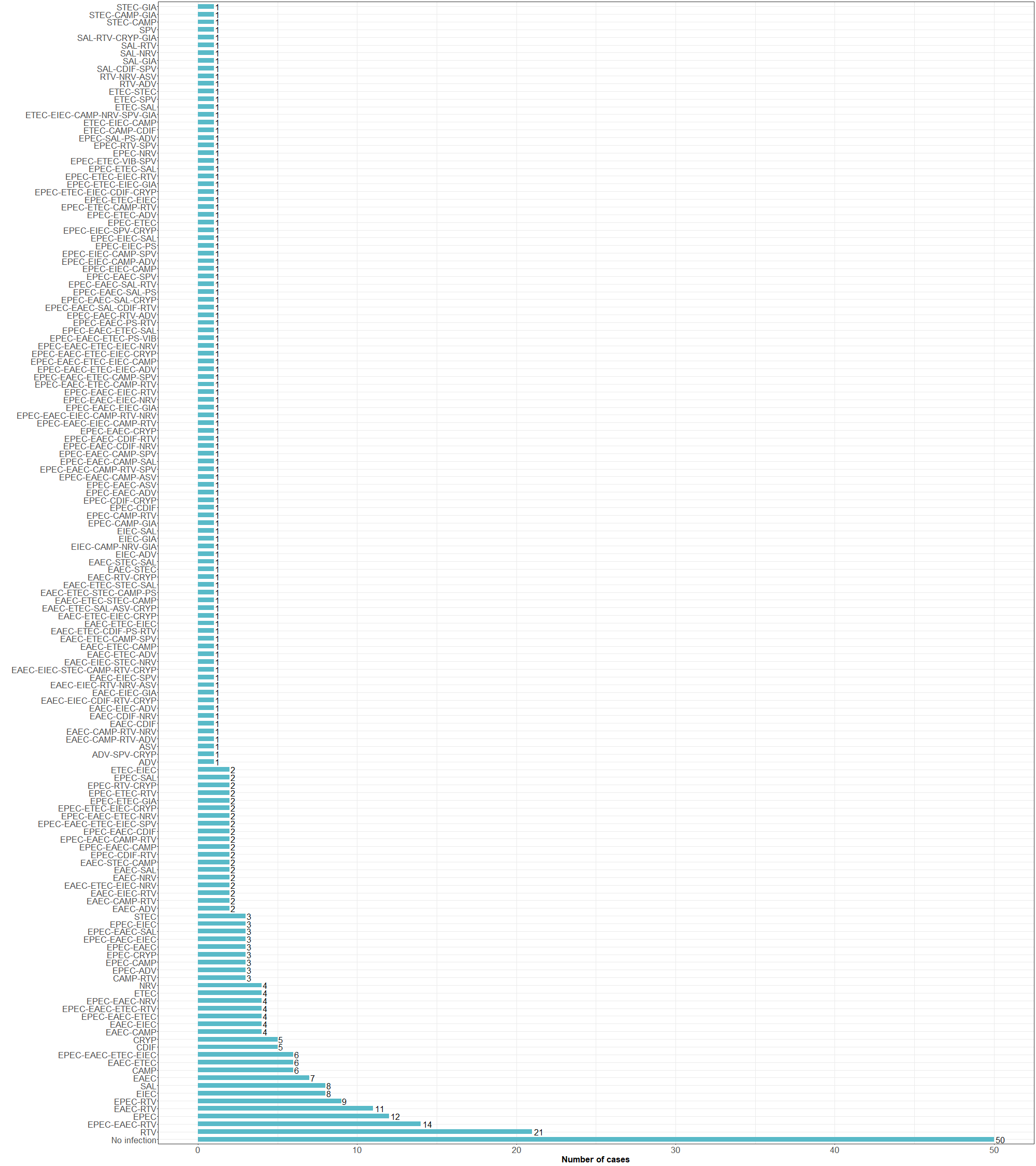

Supplement: S1 Fig — * ADV: Adenovirus F40/41; AST: Astrovirus; CAMP: Campylobacter spp.; CDIF: Clostridium difficile; CRYP: Cryptosporidium spp.; EAEC: Enteroaggregative Escherichia coli; EIEC: Enteroinvasive E. coli/Shigella spp.; EPEC: Enteropathogenic E. coli; ETEC: Enterotoxigenic E. coli; GIA: Giardia duodenalis; NRV: Norovirus GI/GII; PS: Plesiomonas shigelloides; RTV: Rotavirus A; SAL: Salmonella spp.; SPV: Sapovirus (I, II, IV, and V); STEC: Shiga toxin-producing E. coli; VIB: Vibrio cholerae. (TIF) [file pone.0282844.s001.tif]
